# The Irreducibility of Vision: Gestalt, Crowding and the Fundamentals of Vision

**DOI:** 10.3390/vision6020035

**Published:** 2022-06-15

**Authors:** Michael H. Herzog

**Affiliations:** Laboratory of Psychophysics, Brain Mind Institute, École Polytechnique Fédérale de Lausanne (EPFL), 1015 Lausanne, Switzerland; michael.herzog@epfl.ch

**Keywords:** Gestalt theory, crowding, discrete perception, Electroencephalographie (EEG)

## Abstract

What is fundamental in vision has been discussed for millennia. For philosophical realists and the physiological approach to vision, the objects of the outer world are truly given, and failures to perceive objects properly, such as in illusions, are just sporadic misperceptions. The goal is to replace the subjectivity of the mind by careful physiological analyses. Continental philosophy and the Gestaltists are rather skeptical or ignorant about external objects. The percepts themselves are their starting point, because it is hard to deny the truth of one own′s percepts. I will show that, whereas both approaches can well explain many visual phenomena with classic visual stimuli, they both have trouble when stimuli become slightly more complex. I suggest that these failures have a deeper conceptual reason, namely that their foundations (objects, percepts) do not hold true. I propose that only physical states exist in a mind independent manner and that everyday objects, such as bottles and trees, are perceived in a mind-dependent way. The fundamental processing units to process objects are extended windows of unconscious processing, followed by short, discrete conscious percepts.

## 1. A Brief Philosophical Sketch

**Figure 1:**
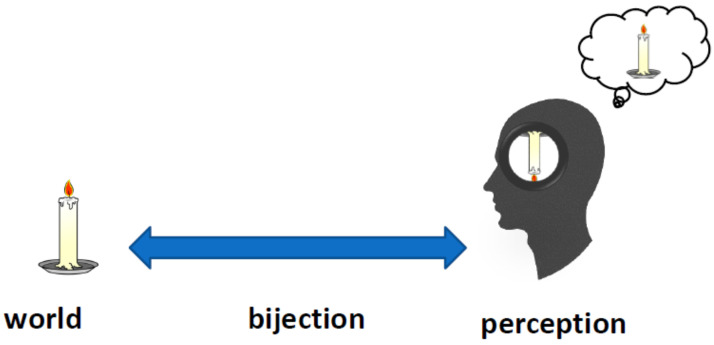
Direct realism: when there is a candle we perceive a candle, and when we perceive a candle, there is a candle out there. It seems there is a 1–1 mapping (bijection) between objects and their mental representations. However, physiology tells us that perception cannot be that direct, since objects are projected upside down and left-right inverted in the eyes.

What a pompous title. What should be fundamental about perception? We open our eyes and see the world as it is. There are objects such as apples and books, whose existence is hard to deny. What can we trust, if not our percepts? This is the position of philosophical direct realism, and many empirical philosophers have, indeed, taken percepts as undeniable ground truth [1,2]. Realism often assumes that there is a 1–1 mapping between the objects of the world and our percepts (Figure 1). When there is a candle in the world we see the candle, and if we see a candle there is a candle out there (leaving aside dreams, mental imagery, and the like).

However, physiology tells us that perception cannot be that direct. Descartes previously noted that there is at least one intermediate processing stage. Because of the laws of optics, the projections of the objects of the external world onto the eye are upside down and left-right inverted. However, we do not perceive objects upside down and left-right. Hence, there must be a re-transformation of the retinal image. Positions like this are called indirect realism. We perceive the world as it is, but not directly: There are intermediate processing stages.

**Figure 2:**
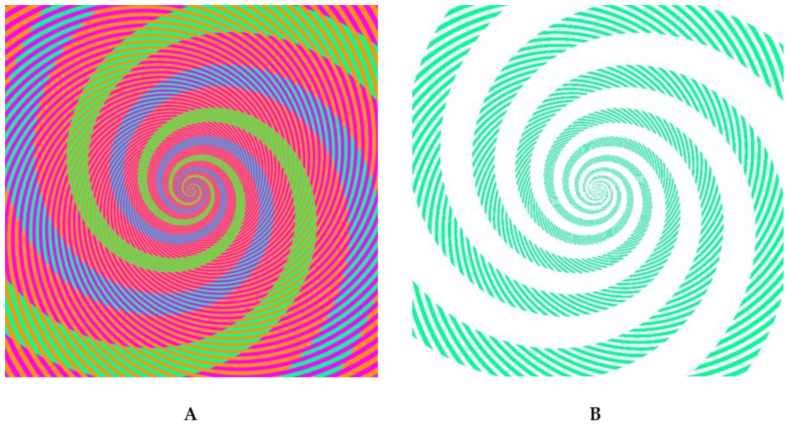
(**A**) Munker-White Illusion; there is no blue. (**B**) All lines removed, except for the green ones. Figures from Akiyoshi Kitaoka [4,5].

Whenever there is a mechanism in nature, there are also failures of this mechanism: when things can go wrong, sometimes they do go wrong. Illusions are such examples, in the case of perception [3]. In Figure 2, for example, most people perceive four colors: blue, green, pink, and red. However, this is one color too many. Which one? One can stare at the figure as long as one wants; introspection does not tell. We perceive four colors in a very stable, indisputable way. However, when determining the colors with a colorimeter, we realize there are, indeed, only three colors and it is the color blue that is missing. One can realize this fact also by removing the red and pink lines, leaving only a white background. Obviously, we can see things (the color blue) that are not out there. Likewise, there are many illusions where there are things out there that we do not perceive, such as in the Lilac Chaser Illusion, where there are disks that appear after starring at them for a while. Hence, there are strange cases where the assumed 1–1 mapping between world objects and perceived objects breaks down. If we cannot always trust our senses, should we trust them then at all? On the other hand, why should we trust colorimeters, which can strongly and systematically fail when badly calibrated? As previously stated, if things can go wrong, sometimes they do so.

Such considerations have led continental philosophers, such as Kant and Fichte, to abandon perception as a source of truth at all. Instead of focusing on external world objects, we should focus on the things we can trust, i.e., the things in our minds. By carefully introspective analysis, we can figure out how our mind works. For example, things are always perceived within a 3D Euclidian space evolving in 1D time. Hence, space time is constitutional for perception and cognition, as we would call it today. Whereas Kant was mainly concerned with cognitive aspects, the phenomenalists re-adopted this program for perception. In particular, Brentano and Husserl tried to work out a science based purely on introspection, i.e., with the percepts as the only source of investigation [6].

## 2. What Is Fundamental in Vision? Gestalt Theory

**Figure 3:**
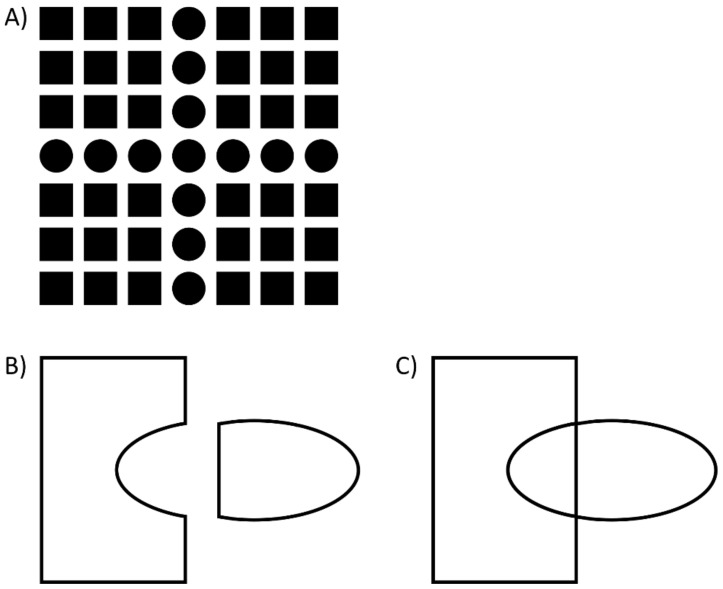
(**A**) Squares and dots are presented, which we can perceive individually. However, there is also an emerging percept of a cross embedded in the squares. (**B**) A piece of a puzzle and a bullet shape are perceived. (**C**) Moving the two objects together produces two new, emerging objects: a rectangle behind an ellipse (or is it the other way around?). When looking only at the left image, it is very hard to imagine what happens when the two shapes are moved to touch each other. Ownership changes: the vertical line of the bullet in (**B**) belongs to the rectangle in (**C**). It is this change in ownership that enables the emergence of the two new objects. From the parts alone, the percept of the whole cannot be predicted. It seems that the whole determines the perception of its parts, and the other way around.

The Gestaltists took these ideas over to marry them with the principles of the natural sciences, i.e., they planted the ideas of phenomenology into the field of empirical testing, with introspection as the key principle. As Gaetano Kanizsa (p. 59 [7]) stated, “The principles of gestalt theory have been discovered and elaborated in the phenomenological realm; it is there that they may be confirmed or rejected…” One of the major discoveries of the Gestaltists was that perception is not fully determined by the visual elements presented. For example, in Figure 3A, a number of squares and dots are presented, of which each can be perceived individually; but there is more. Additionally, we see a cross. This cross is a product of perceptual organization. As Kanizsa said, there is information that goes “beyond the information given” (p. 5, [7]). The goal of the Gestaltists was to understand such types of perceptual organization, which led to the formulation of the well-known Gestalt laws. Kanizsa was a champion in producing extremely compelling demonstrations of the power of these perceptual processes and how they often depend on small changes in the image (ch. 5, [7]). One surprising aspect is that humans cannot predict their own percepts. For example, in Figure 3B, two independent objects are perceived: a piece of a puzzle and a bullet-like figure. What happens if the two are moved towards each other? Two overlapping figures are perceived, a rectangle and an ellipse (Figure 3C), which is almost impossible to predict from Figure 3B. What is even more surprising is that perceptual organization changes the ownership of certain elements. For example, whereas in Figure 3B, the vertical line of the bullet belongs to the bullet, the very same line belongs to the rectangle in Figure 3C. Hence, elements of a display can undergo complex perceptual transformations by simple physical transformations, such as moving elements horizontally onto each other, i.e., without adding elements, etc. The whole determines perception of the parts and vice versa. There are emerging perceptual structures that cannot easily be predicted by the elements making up the structures. This is potentially the main credo of the Gestaltists. Understanding how these structures occur was their agenda.

The Gestalt approach flourished for about two decades, and then more or less disappeared overnight- not because the approach was proven wrong, Gestalt just fell out of fashion and was replaced by a framework that is more or less a resurrection of indirect realism.

**Figure 4:**
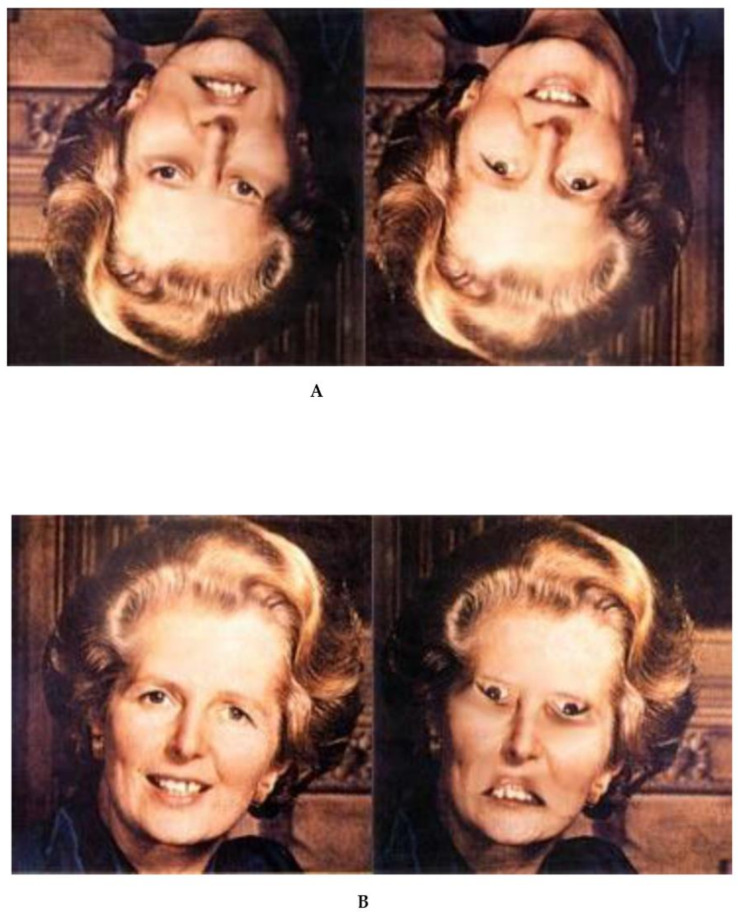
The Margret Thatcher illusion. The holistic oddities are only visible for upright (**B**) but inverted faces (**A**); (from [8]).

I like to add that there are also things out there which we cannot see, even when all the information is given. For example, in the top image in Figure 4, we see two more or less identical inverted faces of former Prime Minister Margret Thatcher. When rotating these faces, we perceive that the right face is quite odd. Closer inspection shows that the mouth and eyes are rotated. While these local differences may also be visible with the inverted faces, the holistic oddity is only visible for the upright faces. 

Summary. In direct realism, the objects of the external are fundamental. Because of the 1–1 mapping, perception is fully determined by the objects. However, there are things we perceive which are not out there (Figure 2). As the Gestaltists have shown, these misperceptions are not funny single cases: the mind systematically goes “beyond the information given” (Figure 3A; p. 5 [7]). Vice versa, there are things out there we cannot perceive (Figure 4). Hence, the 1:1 relationship between objects and percepts seems questionable. Perception cannot deliver ground truth about the external world. In the tradition of continental epistemology, the Gestaltists took the percepts as the fundamental units from which their science started, with introspection as the primary scientific tool. The question of where the objects came from where considered to be rather secondary.

## 3. What Is Fundamental in Vision? The Physiological Framework

### 3.1. The Basic Ideas

As mentioned, for continental philosophy and the Gestaltists, our conscious percepts are crucial, and introspection is the tool of the choice. After World War II, physiological tools became available that allowed a totally different investigation of vision by studying how light affects the eye and the subsequent brain areas. The first decades were mainly concerned with research of the neurophysiology of the retina and the primary visual cortex V1. Benchmark findings were made about phototransduction, neural coding, and receptive fields. Introspection was explicitly not accepted as a tool. The physiological approach can be seen as a causal theory of vision, where mainly the incoming light drives perception.

Computational analysis made clear why perception cannot be direct: perception is genuinely ill-posed. In fact, it turned out that the retina is the worst organ for vision because the light that arrives at the photoreceptors (luminance) is the product of the light shining on an object (illuminance) and the material properties of the object (reflectance): luminance = illuminance × reflectance. The problem is that there are two unknowns (illuminance, reflectance) and only one known (luminance). In principle, there are an infinite number of ways to arrive at one luminance. Hence, from the photoreceptor activity, one can never know whether a strawberry is red and ready to eat or has a different color (reflectance). Were material properties, i.e., reflectance, and illuminance chosen randomly at each point in the visual field, perception would be impossible. However, the illuminance, e.g., the light of the sun or of a light bulb, illuminate large parts of the visual field in a more or less homogenous way. As soon as one knows the illuminance, one can discount for it and determine the reflectance. Almost all processes in vision are ill-posed. Vision, in this framework, subscribes to indirect realism in the sense that there are objects in the world, and the visual brain tries to “reconstruct” these objects by solving the ill-posed problems. The existence of illusions can be seen as a prediction of this framework, since perfect reconstruction might easily fail when the ill-posed problems are hard to solve. Illusions can be seen as a guide to understanding how the brain solves the ill-posed problems.

**Figure 5:**
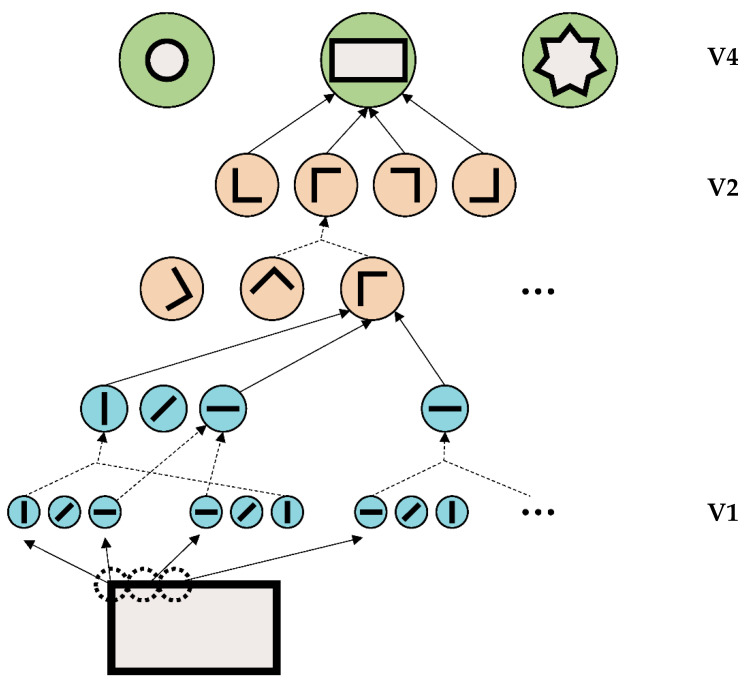
A cartoon of the basic ideas of the physiological framework of vision. For each point in the visual field, there are many neurons in **V1**, each being sensitive to a specific orientation of lines. The outputs of these neurons are pooled to project to neurons in the next processing stage, **V2**. For example, combining the output of edges detectors may create hypothetical neurons sensitive to corners. Such “corner neurons” are active when preceding neurons with the “right” orientation and location are active. Pooling outputs from these corner neurons can give rise to a hypothetical neuron in **V4**, coding for a rectangle.

An important aspect of this framework is its piecemeal and feedforward processing (Figure 5). In the earliest stages of vision, simple features are extracted, such as lines (V1). In higher areas, more and complex features of the objects are computed, such as shapes, or even the face identity of a specific person. These higher representations are fully determined by lower-level processing. Higher-level processing does not influence lower-level processing. This framework makes mathematically perfect sense in the sense that complex problems are broken down into simpler computationally treatable subprocesses. As a side comment: it is well accepted that the visual brain is highly recurrent. However, lateral and recurrent connections are usually attributed to attention and other top-down influences, which are well known to modulate vision.

**Figure 6:**
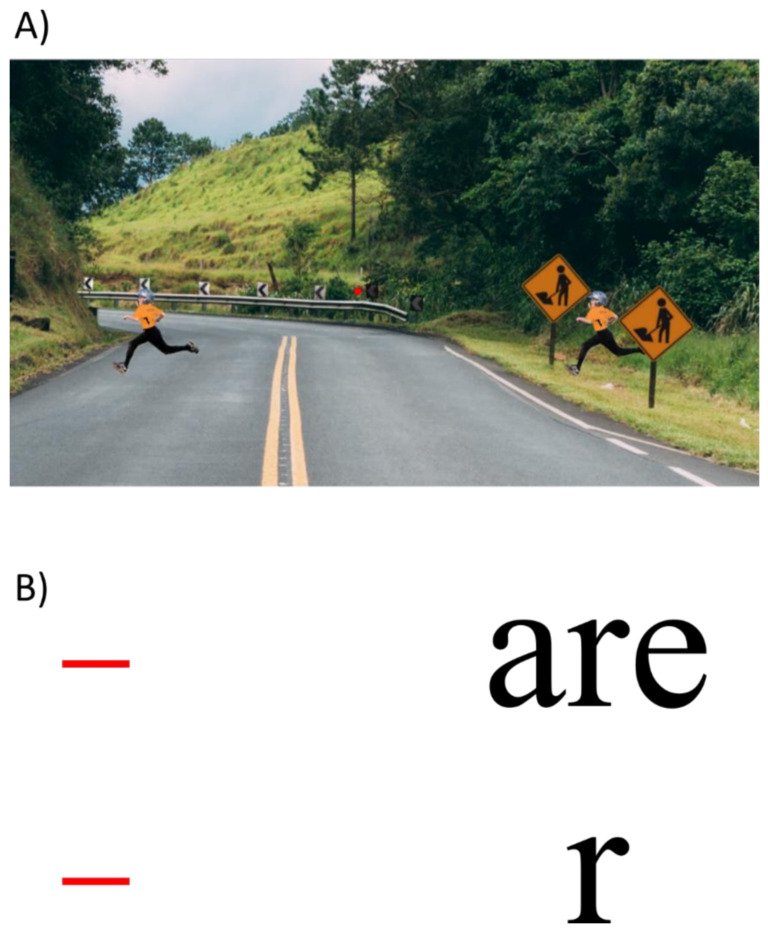
Crowding. (**A**) When staring at the bull′s eye, the child on the left side is easier to detect than the one on the right, crowded by the traffic signs (reproduced from” [9]. (**B**) Usually, crowding is investigated with simple stimuli such as letters. The letter r is hard to detect when flanked by other letters, compared to when presented alone.

For over half a century, the majority of vision research stuck to this framework. For example, psychophysics has tried to exactly determine the basic visual characteristics, such as the channel width of line detectors in V1. In addition, many psychophysical effects could be modeled within this framework. A classic example is crowding. In crowding, the perception of a target strongly deteriorates when presented within clutter or by flanking elements (Figure 6). Crowding is ubiquitous, since elements rarely occur in isolation (except for in psychophysics laboratories). Crowding can easily be explained within this framework because information is pooled from one area to the next to accomplish object recognition. Here is a hypothetical example. A rectangle is presented and processed in the visual hierarchy, beginning with the detection of the edges of the rectangle (Figure 5). In the next step, the outputs of the edge detecting neurons are pooled, “creating” neurons sensitive to corners, which in turn, project to higher-level neurons, coding for a rectangle. Information from one stage to the next is pooled, and thus the receptive field sizes of the neurons increase. In this sense, crowding occurs when object recognition fails because of bad pooling.

This physiological framework has been proven to be computational powerful. For example, deep neural networks, which are well within the spirit of the physiological framework, use exactly the above pooling operations and processing architecture, and exhibit supra-human object recognition. 

### 3.2. Failures of the Physiological Framework

**Figure 7:**
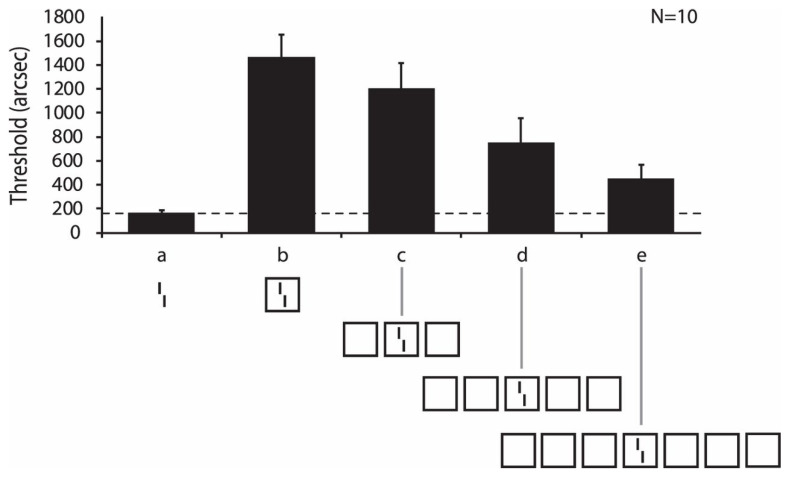
A vernier consists of two vertical lines with the lower line offset either to the left or right, randomly chosen in each trial. Observers indicate the offset direction (a). Performance strongly deteriorates when the vernier is surrounded by the outline of a square (b). Adding squares improves performance, contrary to the prediction of the physiological framework (c–e); reprinted from [16]. Perception of a single element (the vernier) depends on elements extending over 17 deg in this experiment. Performance is plotted as thresholds, where higher values mean worse performance than lower values (contrary to the percentage of correct responses). The hatched line shows the performance of the unflanked vernier.

Whereas the above findings are impressive, the whole framework quickly breaks down when the flanking elements are slightly more complex than those in the classic crowding experiments. For example, we presented a rectangle at 9 deg in the right periphery. First, observers indicated whether the *x*-axis of the rectangle was longer than the *y*-axis. When we flanked the target rectangle with 3 squares on each side, performance strongly deteriorated, a classic crowding effect (results not shown). Next, observers indicated whether a vernier was offset to the left or right. When the vernier was presented within the outline of a square, performance strongly deteriorated, another classic crowding effect. Next, we combined the two conditions (Figure 7). One might expect that crowding further deteriorates because the central square strongly “crowds” the vernier and is itself crowded by the flanking squares. However, this was not the case. Performance reached almost the performance level of the single vernier condition. More flanking elements led to less crowding, contrary to the predictions of most models of crowding (e.g., feedforward pooling: [10,11,12,13,14,15]) and the physiological framework of vision (Figure 5).

**Figure 8:**
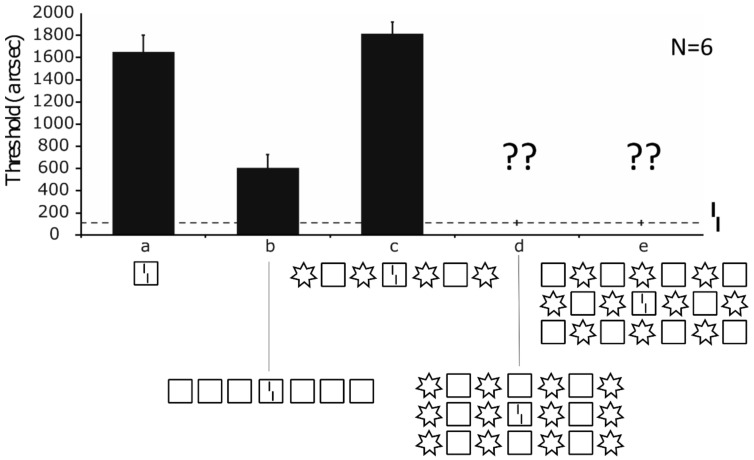
The hatched line shows the performance of the unflanked vernier. As shown before, adding a square strongly deteriorates performance (a), and adding further squares leads to uncrowding (b). Replacing certain squares with stars leads to strong crowding (c). Can you predict what happens if we copy the flanking row above and below (d)? Can you predict what happens when we move the upper and lower row by one inter-element of spacing (e)? The important point of this demonstration is not the actual results, but the impossibility to predict them. The experimental results can be found in [16].

Next, we varied the number of squares. We found a gradual improvement of performance, with worst performance for a single square on each side and the best performance for 2 × 3 squares. Importantly, the vernier offset in the single square condition is about 1–2 arcmin wide. The 7 squares extend over a range of 17° (1020 arcmin), which is more or less the entire right visual field, where vision has good resolution. Hence, the perception of a point in the visual field depends on all the other elements in the visual hemifield. Moreover, the perception depends strongly on the specific stimulus configuration (Figure 8). We proposed that when the target groups with the flankers, crowding is strong. When the target ungroups, uncrowding occurs. These results suggest that we are back in the days of the Gestaltists. The whole determines the perception of its parts as much as the other way around (Figure 3). In addition, we cannot predict the strength of crowding from looking at the stimuli, similar as we cannot predict what happens perceptually when we move the bullet and puzzle piece in Figure 3B,C.

Uncrowding effects are not new [17,18,19,20], but they are ubiquitous: uncrowding does not only occur with vernier stimuli as targets, but also with Gabors [20,21,22,23,24,25,26], letters [27,28], textures [29], complex configurations [30,31], audition [32], and haptics ([33]). Crowding and uncrowding occur both in foveal and peripheral vision, as well as in time [34,35]. As mentioned, except for psychophysics laboratories, (un)crowding is the standard situation in everyday vision, since elements are rarely encountered in isolation. Hence, any theory of vision has to deal with crowding [36,37]. However, no model within the physiological framework even comes close to handling the complex effects presented here, including deep networks, as computer simulations have shown [9,17,38,39,40,41,42]. Only models that took grouping and segmentation explicitly into account and make heavy use of time-consuming recurrent processing delivered good results [9,41,42].

### 3.3. Failures of the Gestaltist Approach 

**Figure 9:**
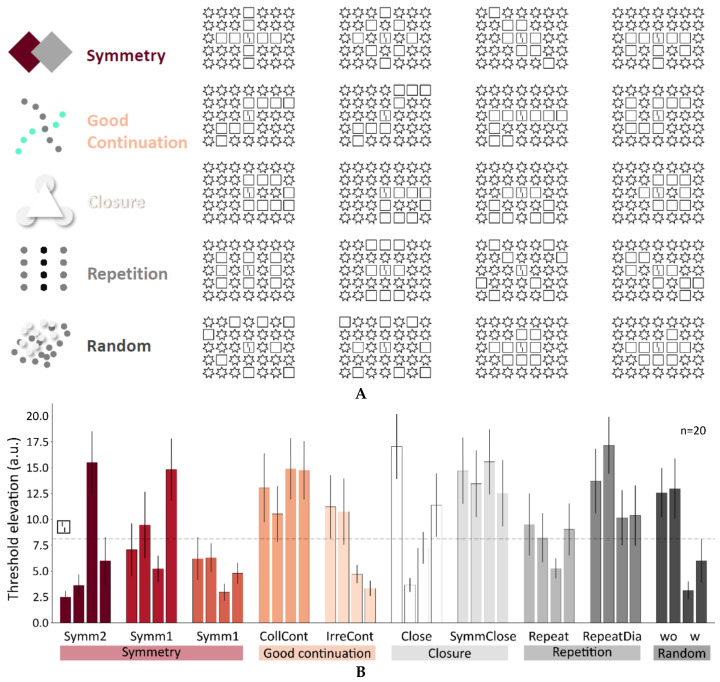
(**A**) We tested many configurations probing the various Gestalt laws, including symmetry (3 types: Symm1–3), good continuation (CollCont, IrreCont), closure (Close, SymmClose), and repetition (Repeat). As a control, we used configurations where star and square positions were randomly chosen (wo, w). In all conditions, there were 9 squares and 25 stars. We tested four stimulus configurations for each type of Gestalt law (40 configurations in total). (**B**) The results show that for each Gestalt law, there are displays for which performance is better than with the single square and others where performance is worse, indicating that the Gestalt laws have very little predictive power (reprinted from [43]).

Next, we tested whether the basic Gestalt laws can explain crowding and uncrowding. The results are quite clear. At least with the stimuli we tested, Gestalt laws fail grossly (Figure 9).

Summary. In the physiological approach, the objects of the external world are the fundamental drivers of perception. There is a causal chain that leads from the reflected light of objects to the perceived objects. Perception is indirect because perception is strongly ill-posed and hence, the features of the objects need to be “reconstructed” (in addition, reconstruction is necessary to cope with missing information caused by retinal blood vessels, the blind spot, and the poor resolution of peripheral vision). Since ill-posed problems cannot be exactly solved, misperceptions, such as in illusions, are not failures of the framework, but can rather provide positive evidence for it. With this approach, the subjective aspects of perception can be eliminated, and science can be objectified.

Low-level processing determines high-level processing, but not the other way around. However, for this reason, this framework can hardly explain why the entire stimulus configuration determines the perception of its parts (Figure 7 and Figure 8). The old Gestalt observation that the whole determines perception of its parts as much as the other way around has no place in this framework. It seems that time-consuming recurrent processing is needed, in which higher-level representations can interact with lower-level ones, and vice versa. Since these processes are time-consuming, they need to be unconscious because we usually do not see how the brain computes them for us. As shown next, there are indeed long-lasting unconscious, recurrent processing periods in vision, which we consider to be fundamental for vision.

## 4. What Is Fundamental in Vision? Time

**Figure 10:**
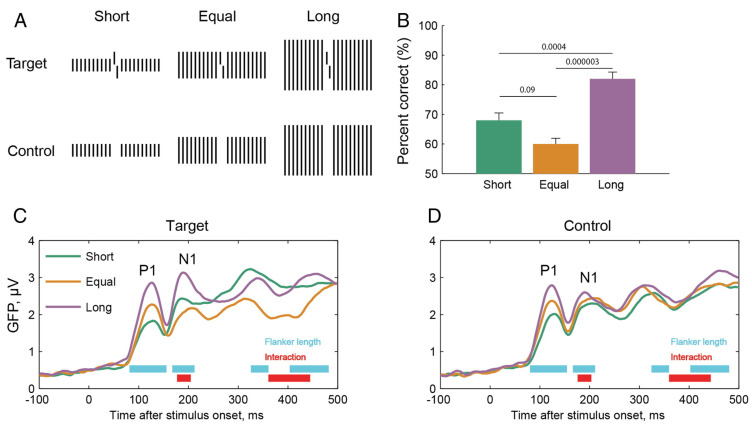
(**A**) Stimuli. A vernier target was presented and flanked by straight lines of the same (yellow), shorter (green), or longer (purple) length. Human observers indicated the vernier offset direction and 128 channel EEG was recorded. Performance is expressed as the percent correct in this experiment. (**B**) Behavioral results. The best performance occurred for the longer lines, intermediate performance for the shorter ones, and worst performance for the equal-length lines (most models of crowding would predict the opposite). (**C**) We measured global field power, which can be taken as a measure of the magnitude of the EEG of all electrodes. About 100–135 ms after stimulus onset, we found the strongest GFP for the long lines, intermediate GFP for the equal length lines, and lowest GFP for the short lines. These results are not surprising, since the P1 is known to reflect low-level stimulus properties, such as the overall luminance. For the N1 at around 176–202 ms, the pattern changes, with higher GFP for the shorter than the equal-length lines. Hence, the N1 predicts crowding strength. We propose that the human brain has transformed a low-level, luminance-based representation unconsciously into a representation reflecting the perceptual organization of the stimuli. (**D**) In the control condition, observers performed no task on the vernier; they just pushed the button they wished. Whereas the P1 amplitudes are as in (**C**), the N1 amplitudes show no reversal in the amplitudes from the shorter to the same length lines suggesting that uncrowding occurs only in a task-dependent, active manner. (**C**) and (**D**) reprinted from ([45]).

Here is an EEG study. We presented a vernier target flanked by arrays of straight lines, i.e., verniers without offset. The flanking lines were either of the same length as the target vernier, longer, or shorter (Figure 10). Performance was best for the longer lines, intermediate for the shorter lines, and worst for the same-length lines. The result is in accordance with the grouping account of crowding: The vernier and the equal-length lines are grouped because all elements have the same length. Most crowding theories would predict the worst performance for the longer lines because there is more “flanker energy”. During the experiment, we recorded 128 channel EEG and computed global field power (GFP). GFP can be seen as a measure of EEG strength, with higher values indicating higher strength. The GFP of the P1 component was highest for the longer lines, intermediate for the equal-length lines, and lowest for the shorter lines. These results are not surprising, since the P1 reflects mainly stimulus energy, and the longer line arrays have the highest overall luminance. For the N1 component, the pattern changed. The highest GFP was again obtained for the longer lines but now, the shorter lines had a higher GFP than the equal-length lines, in accordance with the respective performance levels. The N1 component is known to reflect the spatial layout of stimuli [44]. We suggest that the human brain transforms a basic, luminance-driven representation of the stimuli, reflected by the P1, into an object-based representation, reflected by the N1 [45]. The latter representation determines whether or not crowding occurs (the N1 component is not a direct correlate of performance itself; it reflects the spatial layout, [44]. Hence, there are dynamic processes of which we are not aware, even though processing is a task-dependent, active process (Figure 10D). It seems that we consciously perceive only the output of the processing. 

We propose that the fundamental processes of vision are long-lasting periods of recurrent processing during which the ill-posed problems of vision and many other problems are solved. This unconscious processing has a high spatiotemporal resolution. Consciousness occurs only at discrete moments of time and renders the output of the unconscious processing conscious—contrary to intuition—where perception is continuous. For example, we believe that we see a diver on her trajectory down into the ocean at each moment of time. Our proposal is that we consciously perceive the diver′s motion only at certain moments of time as the output of a motion detector. In this respect, motion and other temporal features are coded in the brain, just as is any other feature, e.g., color and shape. Consciousness renders all these detectors′ values visible for a short moment of time. The following experiment estimates that the unconscious processing periods can last for more than 400 ms.

**Figure 11:**
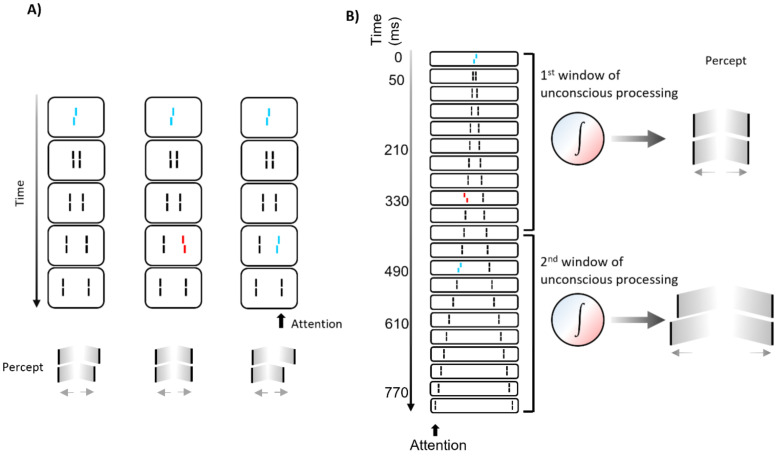
The sequential metacontrast paradigm (SQM). A vernier was presented followed by pairs of flanking lines, which render the vernier unconscious. Observers attend to the left stream. (**A**) left: Even though the vernier is invisible, its offset is perceived at the subsequent lines, which are, in fact, straight. (**A**) middle: When one of the flanking lines is offset in, the two offsets integrate. If the offsets are in opposite directions, they cancel each other. When they are in the same direction, the perceived offset is larger (right). Integration is mandatory, i.e., observers cannot tell which lines are offset. (**B**) Mandatory integration occurs in long-lasting unconscious processing windows. We presented the vernier offset at 0 ms, a line offset in the opposite direction at 330 ms, and another flank offset in the same direction as the vernier at 490 ms. The first two offsets integrated mandatorily, i.e., only the integrated offset could be reported. The third offset could be reported separately, even though the first two offsets were separated by 330 ms and the last two by only 160 ms, i.e., only about twice the time. The last offset did not integrate mandatorily because it is in the subsequent window of processing. All elements were white on a black ground. Colors in this figure are for illustration purposes only. Figure reorganized from [46], with permission.

We presented a vernier followed by a sequence of pairs of flanking straight lines (Figure 11). The vernier is rendered invisible by the flanking lines, as in classic metacontrast masking (where only one pair of lines is presented). Even though the vernier is unconscious, its offset is perceived at the flanking lines, which observers report. When one of the flanking lines was offset as well, this offset and the vernier offset integrated. When both offsets were in the same direction, performance improved. When the offsets were in the opposite direction, performance deteriorated. Importantly, integration is mandatory, i.e., observers cannot tell the offset direction of the vernier or the flanking line separately. They cannot even tell about how many offsets were there. They perceive only one offset, which they report. 

The situation changes when the flanking line offset is presented 490 ms after the vernier offset. Now, both offsets can be reported in a dual report paradigm. Importantly, what matters for integration is belongingness to a temporal window, rather than temporal proximity. When we presented the central vernier, a flank offset in the opposite direction at 330 ms, and a third offset at a flanking line at 490 ms, the first two offsets integrated mandatorily, but not the third one, even though the time between the first two offsets is with 330 ms, about as twice as long as the time between the second and third offset. We propose that the first two verniers fall into one unconscious processing period and hence, integrate. The third offset is in the next unconscious processing period and, hence, does not mandatorily integrate. Thus, there are fundamental processing periods of several hundred milliseconds that determine what we perceive.

Summary: we propose that the fundamental processing units are long-lasting discrete windows, during which the ill-posed problems of vision are resolved in a recurrent and long-lasting manner. This processing is unconscious, and only its output is rendered conscious.

## 5. What Is Fundamental in Vision? Ontology

The Gestalt approach has taken basic percepts, such as the disks and squares in Figure 3, as the fundamental starting point of vision. Introspection provides ground truth. The physiological approach assumes that objects in the external world are the fundamental entities of vision. However, how can such a proposal be justified when perception is not perfect, as is evident in illusions? In addition, it was often shown that humans can perceive the world quite differently (see #theDress). Hence, the old philosophical questions of what is out there are back. If perception is doubtful, is there really nothing else we can start with?

**Figure 12:**
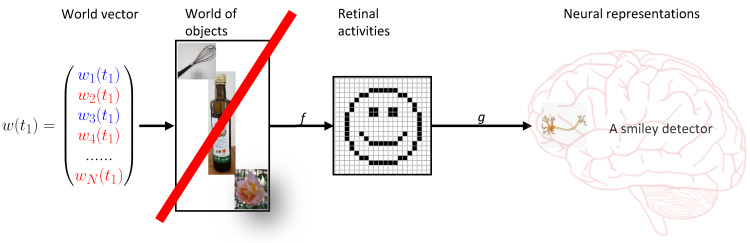
Doubling of ontology. Perception can be seen as a mapping *f* from the world vector to percepts (*g* is the mapping from retina to the mental representations in the brain). Positing an intermediate “world of objects” unparsimoniously doubles the ontology in an unhealthy manner. Physiologically speaking, the mappings for each perceptual system are realized by the individual brain wirings.

Here, I like to argue, there is. As natural scientists, we must believe in the fundamental entities of physics, which are fermion and bosons. We can write the states of the particles as a vector w(t) at time t (Figure 12). Perception is then a mapping from this vector into the set of mental representations, which is much smaller than the number of world states of the vector. Hence, there are many world states that map onto exactly the same mental presentation. Hence, when one perceives an object, one cannot know which state caused the representation of the percept of this object, i.e., the mapping is not invertible. For this reason, vision is not reducible to physics. To keep argumentation as simple as possible, we focus only on states that lead to the very same percept, i.e., the views of one face seen from different angles are different objects.

The crucial point is that the world vector can be partitioned in many ways. Here is a toy example. Assume the world vector has only 4 numbers, which are either 0 or 1. Hence, there are 24 = 16 states. Assume there are two perceptual systems with 4 photoreceptors, and each of these systems has only 2 object representations. For example, perceptual system 1 may map the 8 states with more than 2 zeros on object 1 and the others on object 2. In the perceptual system 2, only (0,0,0,0) and (1,1,1,1) are mapped onto object 1, and the other states are mapped onto object 2. In total, we can create 2^16-1^1 perceptual systems, each having its own partition, i.e., all systems perceive the mini-world differently. Hence, there is no unique way to represent the external world when the number of mental representations is smaller than the number of world states. The world states are mind independent, but the perceived objects are mind-dependent.

There cannot be ground truth for these systems. Both systems map (0,0,0,0) onto object 1, and hence the percepts match. However, for (0,0,0,1), there is no “agreement”. In fact, the percepts are the same in half of the cases and different in the other half, i.e., they are fully uncorrelated.

Both direct and indirect realism assume the existence of a mind independent world of objects in addition to the physical states, which, hence, cannot be true. Realism doubles ontology in an unhealthy, non-unique way: on top of the observer-independent physical states, there would be an observer-dependent world of objects “in between” the physical world and the mental representations of the perceiver (Figure 12). Worse, these objects would depend on the idiosyncrasies of the observer′s perceptual system. Hence, perception is not a mapping from the world vector to the set of objects, followed by a mapping from the set of objects to mental representations. Perception is a direct mapping from the world vector to mental representations., i.e., objects are identical to mental representations.

## 6. What Is Fundamental in Vision? Discussion

Intuitively, it seems that we open our eyes and see the world as it is. There is a 1–1 mapping between the objects of the world and the objects we perceive. However, there are good reasons to abandon the idea of such a mapping. Illusions are one reason. Another one is that the Gestaltists have shown that vision is systematically underdetermined by the stimulus: we see a cross where there are only disks and squares (Figure 3). Hence, the relationship between objects and their mental representations is rather loose. For this reason, perception cannot provide ground truth, as empirically minded philosophers have proposed. Ironically, it is empirical research that does not support empirically minded philosophy. Since the world-perception relationship is rather loose, we now need to answer two questions: what is our fundamental ontology, i.e., what is out there, and what is fundamental in perception?

Following the continental tradition, the Gestaltists took basic percepts as given, such as the squares and disks in Figure 3 (following Kanizsa and the Graz school; see [6]. The primary question was how complex percepts emerge from these basic ones (Figure 3). The question about how these percepts relate to external world objects is important, but of secondary interest. The discovery of a number of Gestalt laws was an important step towards a solution, which, however, was successful only for simple stimulus configurations, where one Gestalt cue prevails (Figure 9). There is the possibility that future research may reveal a fuller picture. However, there are principled reasons that may explain the failure. First, introspection is, by definition, insufficient when, for example, unconscious processes are crucial, for which there is ample evidence (Figure 11). Second, processes, related to the causes of perception, are important for understanding why we see objects. For example, the perception of the cross in Figure 3 may be seen as the perception of an object that is not directly visible because of occlusions and other distortions. In this sense, the cross is not more subjective than the disks; the brain does not add something subjectively, it just tries to infer what is out there (the Gestaltists dismissed inference; p. 5, [6]). The cross is just the best solution to the ill-posed problem of shape, given the sparse information available in the display. Hence, it is important then to know what is out there to understand what needs to be reconstructed. In addition, the specific wiring of the brain is important because it tells us how this reconstruction works. 

With the physiologists, realism is back. There is a world of external objects, which map causally onto mental representations. However, the mapping is strongly distorted, and vision is ill-posed. Hence, the visual system needs to “reconstruct” the objects by systematically restoring them in a piecemeal (feedforward) fashion. Because of the ill-posed problems, one should not be surprised when perception fails under certain circumstances. However, vision is not systematically under-determined. There are no systematic mind-dependent aspects (Figure 3). Quite the contrary; one goal of the approach is to eliminate subjectivity. As the experiments in Figure 7 and Figure 8 show, the physiological approach can explain many results, but quickly breaks down as soon as stimulus configurations become more complex, ruling out all feedforward models in the image in Figure 5. It seems the whole determines the perception of its parts as much as the other way around, in good accordance with the old Gestalt credo. However, we should rather say that the unconscious processing of different elements, such as the vernier target (part) and the array of the squares (whole), interact with each other, leading to certain percepts, respectively. However, these complex interacting, recurrent processes depend very much on the brain wiring, and hence, different wirings may lead to different percepts; thus, subjectivity is back. 

For this reason, we propose that the perception of an object is subjective in the sense that objects are interpretations of the physical world. There is something mind-independent out there, fermions and bosons. However, the objects we perceive are mind-dependent interpretations of the physical world and can be very different between humans and even more so between different species, depending on the brain wirings (Figure 12). Metaphorically, the world is like the words of a poem, which we can all interpret very differently. In the Gestaltist′s notion of subjectivity, the mind adds something subjective that is not out there (the cross) to the things that correspond to what is out there (the dots and squares of Figure 3). We propose that all percepts are subjective: disks, squares, and the cross. The reason for this is that objects are underdetermined in principle because there are more world states than objects, and hence, there can be many mappings. Vision is irreducible because many world states project on the very same objects. We cannot invert the mapping.

To phrase it another way, subjectivity means individuality of perception because of the specific mapping. It does not mean privacy: It may well be that one can infer a percept with neuroimaging. In this sense, we can follow Bishop Berkeley: objects, as condensed interpretations of the physical world, disappear when one closes one′s eyes. However, contrary to Berkeley, the particles of physics stay what they are. They are mind-independent. More information can be found in Herzog and Doerig [47].

So much for ontology. When it comes to visual processing, we propose that the fundamental units of perception are unconscious processing periods, followed by a conscious percept. Perception is a series of these units. We propose that, during these periods, the visual system collects information for several hundreds of milliseconds, with high spatiotemporal resolution, to solve the ill-posed problems of vision, computes motion, and many more time-consuming processes. Only the output of this unconscious processing is rendered conscious (for details, see [48,49]. Unconscious object recognition and motor responses may well occur before consciousness occurs, evident, for example, in fast motor reflexes, which rely on complex image processing, e.g., when grasping something that falls down. 

In summary, the major problem of perception is that we have no good starting point. When we take everyday objects as fundamental, we may simply confuse cause and effect. When we take percepts as the starting point, we miss the cause. It seems that in the Gestalt and physiological approaches, there is a hierarchy of entities: particles->objects->basic percepts->Gestalts. We propose that the chain is much shorter: particles->Gestalts(=objects).
